# The impact of dietary phosphorus levels on growth, slaughter, and digestive metabolism in growing sheep

**DOI:** 10.3389/fvets.2025.1489948

**Published:** 2025-02-06

**Authors:** Shoupei Zhao, Xiaojun Ni, Jia Zhou, Xiaoqi Zhao, Xiao Wen, Xiaolin Wang, Mingyu Cao, Yanfei Zhao, Chong Shao, Lianghao Lu, Yuanyuan Chen, Bao Zhang, Huaming Yang, Bai Xue, Guobo Quan

**Affiliations:** ^1^Institute of Animal Nutrition, Sichuan Agricultural University, Chengdu, China; ^2^Yunnan Animal Science and Veterinary Institute, Kunming, China; ^3^Chongqing Academy of Animal Sciences, Chongqing, China

**Keywords:** sheep, growth performance, slaughtering measurements, metabolism, phosphorus requirement

## Abstract

Phosphorus (P) pollution from livestock farming poses significant environmental challenges, necessitating efficient P utilization. This study systematically investigated the effects of varying dietary P levels on growth, slaughter performance, nutrient digestion, and metabolism in *Yunnan Semi-fine Wool Sheep* during the growth phase. Forty-five sheep (30.33 ± 0.56 kg) were randomly assigned to five dietary P levels (0.40, 0.51, 0.68, 0.82, and 0.97%) over a 44-day trial, including a 14-day pre-feeding and 30-day formal trial period. Digestibility trials were conducted on days 22–27, and selected sheep were slaughtered for detailed analysis. Results showed no significant effects of dietary P on daily weight gain, feed-to-gain ratio, or organ indices (*p* > 0.05). However, dry matter intake, liver, and lung weights decreased linearly with increasing P levels (*p* < 0.05). Carcass traits such as left half carcass rate and net rib rate varied significantly (*p* < 0.05), showing quadratic trends. P levels also affected P, calcium, protein, and energy metabolism, as well as apparent digestibility of acid detergent fiber (*p* < 0.05). Using endogenous loss and comparative slaughter methods, the P maintenance requirement was determined as: Retained *p* = 0.5436 × Intake P – 0.0614 (*R*^2^ = 0.83, *p* < 0.01). P requirements for growth were modeled as: P (g/kg EBW) = 30.95772 × EBW – 0.5031. The recommended dietary P level was 0.40%, with maintenance and growth requirements of 0.06 g/EBW and 5.34–6.19 g/kg EBW, respectively, providing a foundation for P reduction strategies.

## 1 Introduction

Phosphorus (P) is the second most abundant mineral in animals, with approximately 80% stored in bones and teeth, while the remaining portion is distributed across bodily fluids and soft tissues, where it supports critical functions. These include bone formation, energy metabolism, and enzyme activity regulation (1). During the growth phase, P is particularly vital for sheep, playing a pivotal role in skeletal development and maintaining bone density, essential for rapid growth (2). Additionally, P is integral to ATP production and utilization, influencing energy metabolism and enzymatic functions, including protein and nucleic acid synthesis (3, 4). Thus, P is indispensable for optimal growth, energy balance, and biochemical processes in sheep.

Over-application of P in fertilizers and animal feeds has exacerbated environmental concerns, contributing to soil P accumulation and eutrophication of surface waters. While adequate dietary P is essential for physiological functions, over-supplying P beyond the animal’s requirements can lead to environmental challenges. Current estimates suggest that accessible mineral P reserves may be depleted within 50–100 years. Sustainable P management strategies are therefore imperative, focusing on enhancing P utilization efficiency and minimizing P losses from livestock.

The *Yunnan Semi-fine Wool Sheep* is a dual-purpose breed renowned for its adaptability, rapid growth, and utility in both wool and meat production, making it a cornerstone of southwestern China’s livestock industry (5). Despite advancements in understanding P requirements in sheep, these needs vary across breeds, growth stages, and production purposes. Research on P requirements in wool-meat dual-purpose breeds remains limited. According to the Agricultural Research Council (6), the maintenance and growth P requirements for sheep are 0.014 g/(kg BW·d) and 6.0 g/kg EBW, respectively, using the factorial method. The French INRA system recommends 5.5 g/kg BW for growth (7). Bellof and Pallauf (8) found a higher requirement of 7.5 g/kg EBW for German Merino lambs. Similarly, studies on other breeds, such as Ideal × Ile de France and Santa Inês sheep, reported growth P requirements between 4.14 and 5.39 g/kg BW, which are lower than ARC (6) recommendations (9). Domestic research by Ji et al. (10) reported P maintenance and growth requirements of 0.025 g/(kg EBW·d) and 5.27–5.83 g/kg EBW for Dorper × Small-tailed Han sheep. These findings emphasize variability in P requirements among breeds, highlighting the need for targeted research on wool-meat dual-purpose breeds.

The factorial method is widely used to assess animal nutrient requirements, distinguishing between maintenance and growth needs. P maintenance requirements are typically determined via the minimal endogenous loss method, which evaluates basal P excretion under P-free diets (6). However, factors such as feed intake, diet composition, and breed can influence endogenous P losses, requiring further study (11). The comparative slaughter method, in contrast, calculates net growth requirements by assessing P deposition differences pre- and post-experiment (10). This study investigated the effects of varying dietary P levels on growth performance, slaughter characteristics, and nutrient metabolism in *Yunnan Semi-fine Wool Sheep*. Using both the minimal endogenous loss and comparative slaughter methods, it determined P requirements for maintenance and growth, providing insights for optimized feeding strategies and P management practices to enhance production efficiency and reduce environmental impacts.

## 2 Materials and methods

### 2.1 Experimental approval

All experimental procedures involving animals adhered to the Chinese Guidelines for Animal Welfare and received approval from the Ethics Committee of the Yunnan Animal Science and Veterinary Institute (approval number: 202106002). The study was conducted in strict compliance with protocols outlined by the State Science and Technology Commission of the People’s Republic of China (51) and the Standing Committee of the Yunnan Provincial People’s Congress (52).

### 2.2 Animals, experimental design and husbandry practices

Forty-five 10-month-old male *Yunnan Semi-fine Wool Sheep*, with similar body weights (30.33 ± 0.56 kg), were dewormed and randomly assigned to five groups (nine sheep per group). Each sheep was housed individually in separate pens. According to the Chinese standard NY/T 816–2021 (12), the daily phosphorus (P) requirement for meat sheep weighing 30–40 kg is approximately 6.3–7.2 g. However, the requirements of P may differ between meat sheep and dual-purpose sheep (bred for both meat and wool), with limited research available on the latter. In our previous study, the P requirements of non-pregnant ewes of dual-purpose sheep were investigated, with dietary P concentrations ranging from 0.19 to 0.64% (13). A review of phosphorus levels commonly used in related studies (14–19) revealed that typical phosphorus concentrations ranged from 0.30 to 1.01%. Based on this information, and considering dry matter intake (DMI) of the sheep, an appropriate P level for this experiment was determined. To assess the effects of varying phosphorus levels, five experimental diets were formulated with total P concentrations of 0.40, 0.51, 0.68, 0.82, and 0.97%. The levels of P were adjusted by varying the concentrations of sodium dihydrogen phosphate and calcium phosphate across the five groups, while all other nutrient levels were kept consistent across the diets. Details of the diet formulations and nutrient compositions are provided in Table 1.

**Table 1:** Diet composition and nutrient content during the experimental period (% Dry matter basis).

Feed ingredients	Phosphorus level				
	0.40%	0.51%	0.68%	0.82%	0.97%
Corn	26.35	18.53	22.93	23.36	21.05
Bran	9.80	9.88	9.80	9.80	9.80
Soybean meal	2.94	4.20	3.58	3.57	3.98
Sodium dihydrogen phosphate	0.18	0.10	0.08	0.10	0.19
Corn starch	3.25	9.4	5.62	4.85	6.52
Calcium bicarbonate	1.04	0.75	0.40	0.29	0.00
Calcium phosphate	0.00	0.70	1.16	1.60	2.05
Sodium chloride	0.44	0.44	0.43	0.43	0.41
Premix^1^	1.00	1.00	1.00	1.00	1.00
Corn Silage	23.00	23.00	26.00	29.00	30.00
Fava Bean Stick Powder	32.00	32.00	29.00	26.00	25.00
Nutrient content^2^					
ME, MJ/kg	9.42	9.43	9.43	9.42	9.42
CP, %	11.46	11.01	11.61	12.02	12.19
Ca, %	1.46	1.42	1.27	1.38	1.34
P, %	0.40	0.51	0.68	0.82	0.97
NDF, %	29.86	28.02	28.77	27.85	28.38
ADF, %	19.57	19.55	20.10	18.59	19.17

ME, metabolic energy; CP, crude protein; NDF, neutral detergent fiber; ADF, acid detergent fiber; Ca, calcium; P, phosphorus.

1 The premix provided the following per kg of diets: Mn, 58 mg; Fe, 145 mg; Zn, 80 mg; Cu, 10 mg; I, 2.5 mg; Se, 0.35 mg; Co, 0.65 mg; VA, 10,000 IU; VD3, 1,000 IU; and VE, 50 IU.

2 ME is a calculated value, while the other components are measured values.

The experiment lasted 44 days, comprising a 14-day pre-feeding period and a 30-day formal trial. During the pre-feeding period, sheep were acclimated to their housing, feeding regimen, and the experimental diets. Feed amounts were adjusted daily based on the previous day’s intake, ensuring approximately 10% feed remained in the trough. Feeding occurred twice daily at 08:00 and 17:00, with ad libitum access to clean water throughout the experiment. Daily feed intake and leftovers were recorded for each sheep.

### 2.3 Data and sample collection

#### 2.3.1 Growth performance

Each sheep was individually weighed on an empty stomach in the morning on the first and thirtieth days of the experiment. Initial and final body weights were recorded for all individuals. Key performance indicators, such as average daily weight gain (ADG), were calculated using the formula:


$$ADG=\left(\mathrm{Final body weight}\;\left(\mathrm{kg}\right)-\mathrm{Initial body weight}\;\left(\mathrm{kg}\right)\right)/30$$

Daily feed intake and leftover feed for each sheep were also recorded throughout the trial. The DMI was determined by calculating the total feed consumed on a dry matter basis. Additionally, the feed conversion ratio (F/G) was computed as:

$F/G=DMI\;\left(g\right)/ADG\;\left(g\right)$

#### 2.3.2 Digestive metabolism

For the digestive metabolism trials, five sheep from each group were randomly selected between days 22 and 27 of the formal trial period. These sheep were individually housed in metabolic cages to facilitate precise data collection. Daily feces and urine excreted by each sheep were collected, weighed, and recorded over five consecutive days. Concurrently, daily feed intake and feed refusals were also measured and recorded for each sheep to accurately calculate feed utilization. Fresh fecal samples from each sheep were thoroughly homogenized and divided into two 600 g portions. One portion was left untreated to preserve its natural state, while the other portion was treated with 10 mL of 10% sulfuric acid (v/v) per 100 g of fecal sample. This acidification step was performed to stabilize nitrogen for nutrient analysis. All fecal samples were subsequently stored at −20°C until proximate analysis.

#### 2.3.3 Comparative slaughter

At the beginning of the pre-feeding period, one sheep was randomly selected from each group for comparative slaughter to establish a baseline for body composition analysis. At the conclusion of the trial, three sheep per group were randomly chosen for slaughter to assess treatment effects. Prior to slaughter, the selected sheep underwent a 16-h fasting period to standardize the digestive contents. After fasting, the sheep were shorn, and wool samples were collected, weighed, and stored for further analysis.

Slaughter was performed via exsanguination through the jugular vein. Whole blood was collected, weighed, and aliquoted for subsequent analysis of biochemical and hematological parameters. Skinning was then carried out, followed by the systematic dissection of internal organs and the digestive tract. The heart, liver, lungs, kidneys, spleen, and pancreas were carefully separated, weighed, and sampled for analysis. The digestive tract, including the rumen, reticulum, omasum, abomasum, small intestine, and large intestine, was emptied of contents. Each segment was then individually weighed and sampled to evaluate tissue characteristics and nutrient composition. Carcasses were processed to separate muscle and bone. The left half of each carcass was further dissected to record detailed weights, including net muscle, bone, rib, shoulder blade, large bone, and tibia. The organ index was calculated using the following formula:

$\mathrm{Organ index}=\left(\begin{array}{l} \mathrm{Organ weight}\;\left(\mathrm{kg}\right)/Pre-\\ \mathrm{slaughter body weight}\;\left(\mathrm{kg}\right) \end{array}\right)\times 100\%$

### 2.4 Sample laboratory analysis

The content of dry matter (DM, method 930.15), crude protein (CP, method 996.11), organic matter (OM, method 942.05), calcium (Ca, method 985.35), and phosphorus (P, method 986.24) in both feed and feces were analyzed according to the procedures outlined by the Association of Official Analytical Chemists (20). Gross energy (GE) was measured using an automatic adiabatic oxygen bomb calorimeter (Parr 6400 calorimeter, Moline, IL, USA), providing a reliable estimate of the energy content of the samples. Neutral detergent fiber (NDF) and acid detergent fiber (ADF) contents were determined using the methods described by Van Soest et al. (21), employing fiber analysis equipment (Fibertec™ 2010, DEN), which allows for precise measurement of fiber components in the samples. Additionally, the content of calcium (Ca), phosphorus (P), gross energy (GE), and crude protein (CP) in various tissues including urine, skin, wool, bones, meat, viscera, and the digestive tract were also analyzed to assess nutrient distribution and metabolic processes across different body compartments.

The main indicators were calculated as follows:

   Digestible nutrients (g/d) = nutrients intake – fecal nutrients,

   Nutrients apparent digestibility (%) = (digestible nutrients / nutrients intake) × 100,

   Excreted nutrients (g/d) = fecal nutrients + urinary nutrients,

   Retained nutrients (g/d) = nutrients intake – fecal nutrients – urinary nutrients,

   Nutrients retention rate (%) = (retained nutrients/nutrients intake) × 100.

### 2.5 Establishment of phosphorus requirement model for *Yunnan Semi-fine Wool Sheep*

#### 2.5.1 Conversion between body weight and empty body weight

To estimate the P requirement per unit gain in body weight, the ratio of live body weight (BW) to empty body weight (EBW) was used for conversion (22).

#### 2.5.2 The growth requirement of phosphorus

Based on ARC (6), the P content in the body can be derived using a logarithmic allometric growth model with empty body weight (EBW) (Equation 1):


(1) $${\log_{10}}^{\left(y\right)}=a+b\times {\log_{10}}^{\left(x\right)}$$

where y = P mass in the body excluding gut contents (g); a = intercept; b = regression coefficient; x = empty body weight (EBW, kg).

Equation 2 was obtained by differentiating Equation 1 to predict P content at different EBWs:


(2) $$y^{’}=b\times 10^{a}\times {EBW}^{\left(b-1\right)}$$

Where y’ = P requirement per unit increase in empty body weight (g/kg EBW); EBW are in kg; a and b are derived from Equation 1.

#### 2.5.3 The maintenance requirement of phosphorus

P maintenance requirements for *Yunnan Semi-fine Wool Sheep* were determined using a minimal endogenous loss method. P intake and retention were analyzed using linear regression, extrapolating until P intake reached zero to predict the required maintenance P requirement. The intercept of this regression indicates inevitable P losses.

### 2.6 Data statistics and analysis

Experimental data, including production performance, slaughter performance, and digestive metabolism, were analyzed using the PROC MIXED procedure of SAS software (version 9.4, SAS Institute, Cary, NC, USA). Treatments were considered fixed effects, while sheep were treated random effects. The mixed model also included day 0 body weight as a covariate for the analysis of growth performance. Orthogonal polynomial contrasts were employed to evaluate linear, quadratic responses to increasing dietary P levels.

The linear relationship and plots include the relationships between EBW and BW, body P contents and EBW, and P intake and body retained P. These relationships were analyzed using GraphPad Prism’s simple linear regression. Treatment effects were declared significant at *p* < 0.05, with a trend at 0.05 < *p* < 0.1.

## 3 Results

### 3.1 Growth performance

There were no significant differences in the initial weights of the sheep (*p* > 0.05) (Table 2). Furthermore, dietary phosphorus (P) levels did not affect average daily gain (ADG) or final body weight (*p* > 0.05). However, a linear decrease in dry matter intake (DMI) was observed as the dietary P levels increased (*p* = 0.05). Despite this, no significant differences were found in the feed-to-gain ratio (F/G) among the groups (*p* > 0.05).

**Table 2:** Effect of dietary phosphorus levels on growth performance of *Yunnan Semi-fine Wool* Sheep.

Items	Phosphorus level					SEM	*p*-value		
	0.40%	0.51%	0.68%	0.82%	0.97%		Treatment	Linear	Quadratic
IBW, kg	30.48	30.75	30.82	29.95	29.64	1.30	0.96	0.54	0.69
FBW, kg	38.43	37.57	38.28	38.21	37.66	0.49	0.65	0.61	0.82
DMI, kg	1.46	1.38	1.37	1.41	1.29	0.05	0.19	0.05	0.80
ADG, g	271.07	243.74	267.63	260.67	240.85	17.60	0.66	0.47	0.71
F/G	5.42	5.79	5.22	5.57	5.65	0.32	0.75	0.86	0.69

IBW, Initial body weight; FBW, Final body weight; ADG, Average daily weight gain; DMI, Dry matter intake; F/G, Feed intake-to-gain ratio.

^a,b Within a row, means with different superscripts differ, p < 0.05.^

No letter or the same letter superscript indicates a nonsignificant difference (*p* > 0.05), whereas different small letters superscript indicates a significant difference (*p* < 0.05).

### 3.2 Slaughter performance

With increasing dietary phosphorus (P) levels (Table 3), a significant linear decrease in liver and lung indices were observed (*p* < 0.05). However, no significant differences were found among the groups in the indices of the heart, spleen, kidney, and pancreas (*p* > 0.05). In terms of gastrointestinal organs, significant differences were noted in the abomasum index, which showed a linear decrease as dietary P levels increased (*p* < 0.05) (Table 4). However, no significant differences were found in the bone index or other gastrointestinal organ indices across diets with varying P levels (*p* > 0.05). The slaughter rate of *Yunnan Semi-fine Wool Sheep* during the growth period was not significantly influenced by different dietary P levels (*p* > 0.05). Nevertheless, significant differences were observed among the groups in both the left half carcass rate and the net rib rate of the left half (*p* < 0.05). As dietary P levels increased, these measures displayed a linear trend, with the net meat percentage of the left half of the body also showing a linear change (*p* < 0.05). Furthermore, the net rib rate, net shoulder blade rate, and net tibia rate of the left half exhibited a quadratic trend as dietary P levels increased (*p* < 0.05).

**Table 3:** Effect of dietary phosphorus levels on organ index of *Yunnan Semi-fine Wool* Sheep %.

Items	Phosphorus level					SEM	*p*-value		
	0.40%	0.51%	0.68%	0.82%	0.97%		Treatment	Linear	Quadratic
Heart	0.41	0.44	0.40	0.42	0.47	0.03	0.43	0.29	0.26
Liver	1.72	1.70	1.62	1.53	1.49	0.07	0.13	0.01	0.94
Spleen	0.12	0.11	0.12	0.11	0.11	0.01	0.81	0.71	0.46
Lung	1.18	1.16	1.00	1.05	1.03	0.05	0.09	0.03	0.19
Kidney	0.25	0.22	0.24	0.22	0.23	0.01	0.32	0.46	0.53
Pancreas	0.0025	0.0033	0.0025	0.0023	0.0025	0.0005	0.68	0.52	0.86

^a,b Within a row, means with different superscripts differ, p < 0.05.^

No letter or the same letter superscript indicates a nonsignificant difference (*p* > 0.05), whereas different small letters superscript indicates a significant difference (*p* < 0.05).

**Table 4:** Effect of dietary phosphorus levels on slaughter performance of *Yunnan Semi-fine Wool* Sheep %.

Items	Phosphorus level					SEM	*p*-value		
	0.40%	0.51%	0.68%	0.82%	0.97%		Treatment	Linear	Quadratic
Organ index									
Bone rate	11.53	9.48	10.92	11.12	11.34	0.66	0.26	0.48	0.30
Esophageal index	0.08	0.10	0.10	0.12	0.13	0.02	0.52	0.10	0.83
Rumen index	1.77	1.72	1.70	1.65	1.88	0.12	0.71	0.70	0.25
Reticulum index	0.31	0.28	0.26	0.22	0.30	0.05	0.12	0.26	0.03
Omasum index	0.31	0.25	0.27	0.26	0.25	0.22	0.27	0.14	0.54
Abomasum index	0.72^a^	0.55^bc^	0.64^ab^	0.45^c^	0.53^bc^	0.04	0.01	<0.01	0.24
Intestine index	3.23	3.56	3.54	3.46	3.74	0.24	0.68	0.27	0.91
Slaughter performance									
Slaughtering rate	47.96	45.10	48.61	49.51	48.53	1.08	0.11	0.11	0.95
Left half carcass slaughter rate									
Left half carcass rate	23.74^a^	19.77^b^	24.23^a^	24.46^a^	24.61^a^	1.01	0.03	0.05	0.55
Net meat percentage of the left half of the body	18.73	15.91	19.34	20.05	19.93	0.94	0.06	0.04	0.71
Net bone percentage of the left half of the body	4.42	3.62	4.17	4.09	4.28	0.30	0.45	0.76	0.35
Net rib rate of the left half of the body	0.41^bc^	0.31^c^	0.39^bc^	0.42^b^	0.55^a^	0.04	0.01	<0.01	0.02
Net shoulder blade rate of the left half of the body	0.23	0.17	0.17	0.19	0.21	0.02	0.16	0.63	0.03
Net large bone percentage of the left half of the body	2.32	1.94	2.36	2.21	2.07	0.25	0.73	0.83	0.81
Net tibia rate of the left half of the body	1.45	1.21	1.25	1.27	1.45	0.07	0.09	0.67	0.01

^a,b Within a row, means with different superscripts differ, p < 0.05.^

### 3.3 Ash, P, and Ca content in the organs

There were no differences observed in the P, Ca, and Ash content of wool, hide, meat, and bones across all treatment groups (*p* > 0.05) (Table 5) suggesting that dietary treatments had no influence on these above parameters.

**Table 5:** Effect of dietary phosphorus levels on slaughter performance of *Yunnan Semi-fine Wool* Sheep %.

Items	Phosphorus level					SEM	*p*-value		
	0.40%	0.51%	0.68%	0.82%	0.97%		Treatment	Linear	Quadratic
Wool Ash	6.24	6.18	5.74	5.70	7.50	1.71	0.94	0.71	0.52
Wool P	0.0367	0.0403	0.0262	0.0424	0.0302	0.0073	0.51	0.62	0.99
Wool Ca	0.2012	0.1856	0.1946	0.1886	0.1885	0.0087	0.72	0.47	0.71
Sheepskin Ash	0.0734	0.0810	0.0722	0.0834	0.0894	0.0053	0.20	0.07	0.38
Sheepskin P	0.0075	0.0065	0.0061	0.0069	0.0073	0.0007	0.57	0.89	0.14
Sheepskin Ca	0.0027	0.0024	0.0023	0.0032	0.0028	0.0002	0.12	0.17	0.54
Mutton Ash	0.1356	0.1252	0.1369	0.1441	0.1524	0.0116	0.57	0.17	0.54
Mutton P	0.0242	0.0219	0.0262	0.0256	0.0246	0.0017	0.47	0.38	0.57
Mutton Ca	0.0072	0.0058	0.0076	0.0079	0.0068	0.0006	0.16	0.46	0.45
Bone Ash	0.3773	0.2778	0.3175	0.4086	0.3703	0.0079	0.78	0.62	0.65
Bone P	0.0731	0.0502	0.0581	0.0681	0.0646	0.0124	0.73	0.93	0.47
Bone Ca	0.1478	0.1000	0.1157	0.1488	0.1274	0.0287	0.71	0.88	0.62

Ash, crude ash; P, phosphorus; Ca, calcium.

^a,b Within a row, means with different superscripts differ, p < 0.05.^

### 3.4 Digestion coefficients

As dietary phosphorus (P) levels increased (Table 6), significant differences were observed in P intake, fecal P, digestible P, apparent P digestibility, excreted P, retained P, and P retention rate (*p* < 0.01). These parameters all showed a linear increase as dietary P levels increased except P retention rate (*p* < 0.01). At different dietary P levels (Table 7), no significant differences in calcium (Ca) intake were found among the groups (*p* > 0.01). However, significant differences were observed in urine Ca, fecal Ca, Ca apparent digestibility, excreted Ca, and Ca retention rate (*p* < 0.05). All indicators, except urinary Ca, showed a linear increase (*p* < 0.01). As dietary P levels increased (Table 8), significant differences were observed in fecal crude protein (CP) content among the groups (*p* = 0.05), showing a linear decrease (*p* < 0.05). Additionally, a quadratic decline in CP apparent digestibility and CP retention rate was noted (*p* < 0.05). With increasing dietary P levels (Table 9), a linear decrease in fecal gross energy (GE) excretion was observed, while a linear increase in the apparent digestibility and deposition rates of GE was noted (0.05 < *p* < 0.1). As dietary P levels increased (Table 10), fecal acid detergent fiber (ADF) content exhibited a linear variation (*p* < 0.05), while no significant differences were observed among the groups in neutral detergent fiber (NDF) metabolism (*p* > 0.05). The dietary P level did not affect the digestibility of ash (Table 11), and no significant differences were found among the groups in terms of fecal ash excretion or ash apparent digestibility (*p* > 0.05).

**Table 6:** Effect of dietary phosphorus levels on phosphorus metabolism of *Yunnan Semi-fine Wool* Sheep.

Items	Phosphorus level					SEM	*p*-value		
	0.40%	0.51%	0.68%	0.82%	0.97%		Treatment	Linear	Quadratic
P intake, mg	5919.06	7346.50	8410.08	12,454	13,705	430.18	<0.01	<0.01	0.24
Urine P, mg	10.23	17.05	15.29	8.47	8.74	2.75	0.12	0.17	0.14
Fecal P, mg	4505.54^d^	5442.44^c^	7161.48^b^	8178.79^a^	8451.26^a^	309.80	<0.01	<0.01	0.04
Digestible P, mg	1413.53^b^	1904.07^b^	1248.62^b^	4274.84^a^	5253.40^a^	389.97	<0.01	<0.01	<0.01
P apparent Digestibility, %	23.52	25.92	11.87	34.25	38.42	4.18	<0.01	<0.01	0.02
Excreted P, mg	4515.77^d^	5459.48^c^	7176.77^b^	8187.26^a^	8460.00^a^	310.13	<0.01	<0.01	0.04
Retained P, mg	1403.29^b^	1887.01^b^	1233.32^b^	4266.36^a^	5244.73^a^	389.53	<0.01	<0.01	<0.01
P retention rate, %	23.52^bc^	25.92^b^	11.87^c^	34.25^a^	38.42^a^	4.18	<0.01	<0.01	0.02

P, phosphorus.

^a,b Within a row, means with different superscripts differ, p < 0.05.^

**Table 7:** Effect of dietary phosphorus levels on Ca metabolism of *Yunnan Semi-fine Wool* Sheep.

Items	Phosphorus level					SEM	*p*-value		
	0.40%	0.51%	0.68%	0.82%	0.97%		Treatment	Linear	Quadratic
Ca intake, g	21.87	20.35	19.75	21.01	18.90	0.81	0.14	0.06	0.89
Urine Ca, g	0.03^b^	0.04^b^	0.14^a^	0.06^b^	0.04^b^	0.02	<0.01	0.48	<0.01
Fecal Ca, g	14.20^a^	14.19^a^	13.09^a^	12.98^a^	11.30^b^	0.52	<0.01	<0.01	0.30
Digestible Ca, g	7.67	6.16	6.66	8.03	7.60	0.63	0.23	0.35	0.30
Ca apparent Digestibility, %	35.00^bc^	30.31^c^	32.88^bc^	38.24^b^	40.25^a^	2.20	0.03	0.01	0.10
Excreted Ca, g	14.23^a^	14.23^a^	13.23^a^	13.04^a^	11.35^b^	0.53	<0.01	<0.01	0.25
Retained Ca, g	7.64	6.11	6.52	7.97	7.56	0.63	0.21	0.36	0.25
Ca retention rate, %	34.86	30.08	32.17	37.95	40.01	2.22	0.03	0.01	0.12

Ca, calcium.

^a,b Within a row, means with different superscripts differ, p < 0.05.^

**Table 8:** Effect of dietary phosphorus levels on Ca metabolism of *Yunnan Semi-fine Wool* Sheep.

Items	Phosphorus level					SEM		*p*-value	
	0.40%	0.51%	0.68%	0.82%	0.97%		Treatment	Linear	Quadratic
CP intake, g	171.33	158.32	163.58	182.56	172.21	6.47	0.13	0.19	0.59
Urine CP, g	17.24	21.36	21.08	21.05	22.66	1.96	0.40	0.20	0.02
Fecal CP, g	84.34^a^	84.19^a^	83.23^a^	86.24^a^	71.20^b^	3.62	0.05	0.04	0.06
Digestible CP, g	86.99^ab^	74.12^b^	80.35^b^	96.32^a^	101.01^a^	5.32	0.01	<0.01	0.06
CP apparent Digestibility, %	50.75^bc^	46.92^c^	48.49^b^	52.76^b^	58.66^a^	1.87	<0.01	<0.01	<0.01
Excreted CP, g	101.58	105.56	104.31	107.29	93.86	4.44	0.27	0.32	0.08
Retained CP, g	69.75^ab^	52.74^b^	59.26^b^	75.26^a^	78.44^a^	4.51	<0.01	<0.01	0.02
CP retention rate, %	40.77^ab^	33.36^c^	35.67^bc^	41.22^ab^	45.54^a^	2.11	<0.01	<0.01	<0.01

CP, Crude protein.

^a,b Within a row, means with different superscripts differ, p < 0.05.^

**Table 9:** Effect of dietary phosphorus levels on GE metabolism of *Yunnan Semi-fine Wool* Sheep.

Items	Phosphorus level					SEM	*p*-value		
	0.40%	0.51%	0.68%	0.82%	0.97%		Treatment	Linear	Quadratic
GE intake, MJ	14.07	13.55	13.70	14.30	13.30	0.53	0.68	0.60	0.81
Urine GE, MJ	0.31	0.27	0.31	0.32	0.32	0.02	0.28	0.27	0.42
Fecal GE, MJ	10.09	9.89	9.46	9.81	8.68	0.46	0.25	0.06	0.54
Digestible GE, MJ	3.98	3.66	4.24	4.49	4.61	0.48	0.63	0.19	0.85
GE apparent digestibility, %	28.15	27.09	30.34	31.33	34.73	2.93	0.41	0.07	0.61
Excreted GE, MJ	10.40	10.16	9.77	10.13	9.00	0.47	0.28	0.07	0.56
Retained GE, MJ	3.67	3.39	3.93	4.17	4.30	0.48	0.68	0.19	0.87
GE retention rate, %	25.96	25.11	28.07	29.08	32.34	3.00	0.48	0.09	0.64

GE, gross energies.

^a,b Within a row, means with different superscripts differ, p < 0.05.^

No letter or the same letter superscript indicates a nonsignificant difference (*p* > 0.05), whereas different small letters superscript indicates a significant difference (*p* < 0.05).

**Table 10:** Effect of dietary phosphorus levels on NDF and ADF metabolism of *Yunnan Semi-fine Wool* Sheep.

Items	Phosphorus level					SEM	*p*-value		
	0.40%	0.51%	0.68%	0.82%	0.97%		Treatment	Linear	Quadratic
NDF intake, g	446.34	402.78	411.74	422.89	400.77	16.11	0.29	0.21	0.56
Fecal NDF, g	328.10	302.58	297.09	321.04	280.64	14.25	0.17	0.11	0.91
Digestible NDF, g	118.24	100.20	114.65	101.85	120.13	14.39	0.79	0.86	0.44
NDF apparent Digestibility, %	26.34	25.06	27.22	24.02	29.96	3.00	0.68	0.50	0.43
ADF intake, g	292.59	281.02	287.43	282.26	270.78	11.06	0.71	0.25	0.74
Fecal ADF, g	221.47	202.54	202.82	206.20	182.61	8.58	0.07	0.01	0.77
Digestible ADF, g	71.12	78.48	84.48	76.07	88.17	9.16	0.70	0.29	0.90
ADF apparent Digestibility, %	24.13	28.13	28.75	26.90	32.53	2.64	0.29	0.08	0.88

NDF, Neutral detergent fiber; ADF, Acid washing fiber.

^a,b Within a row, means with different superscripts differ, p < 0.05.^

No letter or the same letter superscript indicates a nonsignificant difference (*p* > 0.05), whereas different small letters superscript indicates a significant difference (*p* < 0.05).

**Table 11:** Effect of dietary phosphorus levels on Ash metabolism of *Yunnan Semi-fine Wool* Sheep.

Items	Phosphorus level					SEM	*p*-value		
	0.40%	0.51%	0.68%	0.82%	0.97%		Treatment	Linear	Quadratic
Ash intake, g	112.97	108.34	107.83	117.49	110.85	4.23	0.50	0.69	0.79
Fecal Ash, g	69.92	67.01	68.82	72.11	63.12	3.17	0.37	0.42	0.31
Digestible Ash, g	99.92	89.48	90.37	106.50	93.88	10.29	0.75	0.86	0.86
Ash apparent digestibility, %	87.59	81.99	81.14	90.55	84.38	6.17	0.80	0.89	0.76

Ash, crude ash.

^a,b Within a row, means with different superscripts differ, p < 0.05.^

### 3.5 Phosphorus requirement modeling

**Figure 1:**
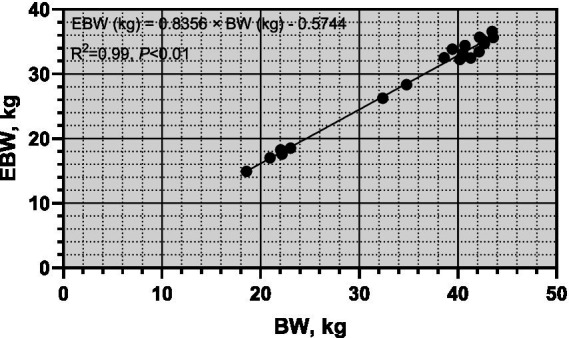
Linear relationship between empty body weight and Pre-slaughter body weight of growing *Yunnan Semi-fine Wool* Sheep. BW, body weight; EBW, empty body weight.

**Figure 2:**
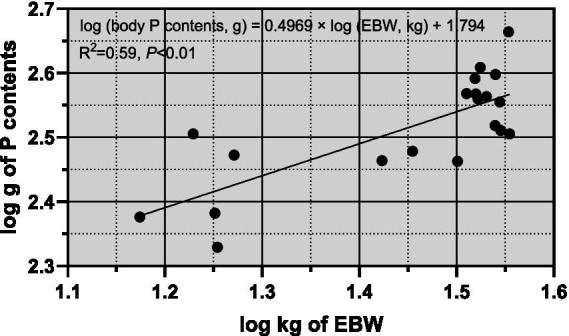
Relationship between body P contents and EBW of growing *Yunnan Semi-fine Wool* Sheep. P, phosphorus; BW, body weight; EBW, empty body weight.

From Figure 1, it was observed that there was a significant linear correlation (*R*^2^ = 0.99, *p* < 0.01) between empty body weight (EBW) and pre-slaughter live weight (BW), suggesting that BW can be reliably used to predict EBW in sheep. Figure 2 illustrates the allometric regression relationship between mineral content in the body and EBW. The equation for predicting phosphorus (P) content based on EBW was: log (body P contents, g) = 0.4969 × log (EBW, kg) + 1.794, with an *R*^2^ of 0.59 and *p* < 0.01. This equation allows predictions of P content per unit of EBW. Using this relationship, the P requirement for growth in *Yunnan Semi-fine Wool Sheep* was estimated as follows:

$P\;\mathrm{requirement for growth},g/\mathrm{kg}\;EBW=30.95772\times {EBW}^{-0.5031}$

**Figure 3:**
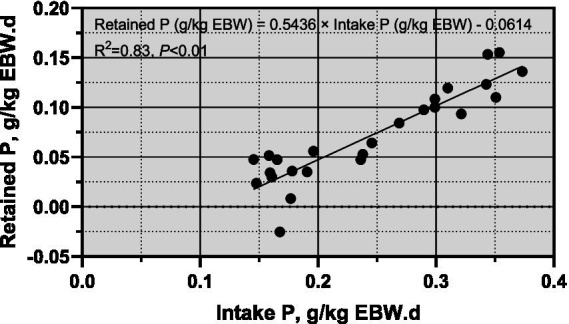
Relationship between Phosphorus intake and body retained P growing *Yunnan Semi-fine Wool* Sheep. P, phosphorus; BW, body weight; EBW, empty body weight.

For sheep weighing between 30 and 40 kg, the P requirements ranged from 6.19 to 5.34 g/kg EBW. Figure 3 showed a significant linear relationship between P intake (g/kg EBW) and P deposition (g/kg EBW). The regression equation was: Retained P (g/kg EBW) = 0.5436 × Intake P (g/kg EBW) – 0.0614, with an *R*^2^ of 0.83 and *p* < 0.01. The intercept of the regression equation represents the endogenous and metabolic losses of P, interpreted as the net mineral maintenance requirements. Therefore, the P maintenance requirement of *Yunnan Semi-fine Wool Sheep* during the growth period was estimated to be 0.06 g/EBW.

## 4 Discussion

Phosphorus (P) requirements in livestock are influenced by factors such as breed, growth stage, production purposes, and environmental conditions (23). Specifically, for the *Yunnan Semi-fine Wool Sheep*, a dual-purpose breed used for both wool and meat production, research on its P requirements is lacking, particularly during key growth phases. In the present study, increasing dietary P levels resulted in a trend toward a linear decrease in feed intake, although the differences observed were not statistically significant. This finding contrasts with previous studies on dairy cows, which reported no significant effect of P levels on feed intake, likely due to the diets being within normal P intake ranges (24, 25). The results of our study suggest that insufficient dietary P may fail to meet the nutritional needs of growing sheep, prompting an increase in feed intake to improve P uptake. However, no significant effects on daily weight gain or feed conversion ratio were observed, consistent with earlier studies (25, 26).

There is limited research examining the effects of P on organ indices and slaughter performance in sheep. In our experiment, higher dietary P levels tended to reduce liver and lung indices, although the differences were not statistically significant. Some studies suggest that excess dietary P can influence the regulation of parathyroid hormone (PTH) and fibroblast growth factor-23 (FGF-23), which could potentially contribute to the increased burden on organs such as the liver and lungs, suggesting a risk of organ damage from elevated P levels (27). Phosphorus absorption primarily occurs in the form of inorganic phosphate salts, with the majority of P being absorbed through the intestine, with a smaller portion absorbed through the stomach (28). When animals consume low-P diets, absorption is facilitated by a calcium-independent active transport mechanism, whereas when P intake meets or exceeds requirements, absorption predominantly occurs passively.

In our experiment, increased dietary P levels led to a reduction in the rumen index, especially at P levels of 0.82 and 0.97%. Studies on cows with right-sided displaced abomasum or abomasal torsion often indicate hyperphosphatemia, potentially associated with dehydration and reduced renal blood flow (29), suggesting a possible link between high P levels and a reduction in rumen index. These results warrant further investigation. The impact of dietary P levels on slaughter performance has been inconsistent in previous studies. Research on poultry has shown that low P levels can reduce carcass weight (30), while studies on larger animals, such as pigs and sheep, have found no significant effects (31, 32). In our study, dietary P levels did not significantly affect slaughtering rate. However, further analysis revealed that the left-side carcass slaughter rate was lowest at the 0.51% P level, with a linear increase observed as dietary P levels increased, except for the lowest group. This suggests that P levels play a role in influencing the slaughter performance of growing *Yunnan Semi-fine Wool Sheep*.

Phosphorus is critical for maintaining skeletal health (33). As a component of inorganic phosphate, P is essential for bone development and mineralization, particularly during growth and post-pubertal bone strength maintenance (34). These findings are consistent with our study, which observed a linear increase in the net rate of the left rib as P levels increased from 0.51 to 0.97%. However, no significant effects were noted on the calcium (Ca), P, or ash content in the bone. These findings align with previous studies showing that P supplementation in grazing sheep can increase bone and muscle mass without significantly altering protein, fat, P, or ash content in bone (31). Similarly, studies on dairy cows have indicated that low dietary P does not affect the Ca, P, or ash content in the coccygeal vertebrae (35). In poultry, P-deficient diets have been shown to reduce tibial bone density and fracture strength (36), and similar effects have been reported in goslings (37). Some inconsistencies may arise from species-specific differences and the ratio of dietary Ca to P.

While the effect of dietary P on digestive metabolism in sheep is underexplored, our study examined the impact of varying P levels on the digestion and metabolism of P, Ca, energy, crude protein, neutral detergent fiber (NDF), acid detergent fiber (ADF), and ash. As expected, increasing P intake led to a linear increase in fecal P excretion, consistent with findings from Feng et al. (38) and Puggaard et al. (39). However, urinary P excretion did not vary with increasing dietary P, although total P excretion exhibited a linear increase. Both apparent digestibility and retention rates of P increased with higher dietary P, except in the 0.68% group, where lower dry matter intake reduced P intake without a corresponding decrease in total P excretion (40). Some studies have questioned the role of vitamin D in regulating P absorption, suggesting that absorption efficiency may be largely independent of dietary P levels, with variations in fecal P excretion being attributed to changes in salivary inorganic phosphate secretion (41). In our experiment, no significant differences were observed between the 0.82 and 0.97% groups, which may reflect a saturation of the passive transport mechanism at high P intake levels (38).

The fecal excretion of Ca decreased as dietary P levels increased, which is consistent with earlier studies. P deficiency reduces Ca absorption in the intestines, leading to a decrease in plasma 1,25-dihydroxyvitamin D concentration (42). As a result, both the apparent digestibility and deposition rates of Ca increased quadratically with increasing dietary P, with the lowest values observed at the 0.51% P level. The enhanced Ca digestibility and deposition in the 0.40% P group may reflect compensatory mechanisms, leading to increased dry matter intake. Additionally, the higher ratio of dietary Ca to P relative to fecal Ca to P indicates greater intestinal Ca absorption in the hindgut fermentation vessel (43). The NRC also suggests a strong correlation between nitrogen and P content in growing pigs, indicating mutual interrelations in their deposition (44).

P affects nitrogen metabolism and utilization in cattle. Studies in cattle have shown that insufficient P intake impairs rumen fermentation, reducing microbial protein production and nutrient digestibility (45). Experiments *in vitro* of cattle also demonstrate that inadequate P supply negatively impacts total rumen bacteria, specific microbial species, and fiber degradation (46). Our study supports these findings, with increased dietary P improving nitrogen digestibility and reducing nitrogen excretion. Therefore, enhancing the apparent digestibility and deposition rate of crude protein.

As P is a critical component of energy-producing reactions, playing a role in the formation of high-energy phosphate bonds such as adenosine triphosphate (ATP), it is essential for energy metabolism (47). In our study, increasing dietary P levels was associated with a linear decrease in fecal energy excretion and a linear increase in energy deposition, highlighting the role of P in regulating metabolic processes. The effect of dietary phosphorus (P) levels on ADF digestibility was similarly observed in our experiments. As dietary P levels increased, a linear reduction in fecal ADF content was noted, accompanied by a clear improvement in ADF digestibility. However, no effect of dietary P levels on NDF digestion was detected.

The P maintenance and growth requirements of *Yunnan Semi-fine Wool Sheep* were determined through metabolic and comparative slaughter experiments. Using the minimum endogenous loss method, we estimated the P maintenance requirement for these sheep during the growth phase to be 0.06 g/EBW. The ARC (53) set the P maintenance requirement for sheep at 42.5 mg/kg BW, later adjusting this to 14 mg/kg BW in the 1980 recommendations, translating to 0.6–0.8 g in our experimental conditions. The NRC (54) recommended a P maintenance requirement of 20 mg/kg BW for growing sheep, which is a 28.6% increase over the ARC (6) recommendation. Studies in China suggested a maintenance requirement of 0.03 g/kg EBW for sheep weighing 25–35 kg, which corresponds to 0.73–0.99 g for sheep in the 30–40 kg range. The INRA (55) recommended 30 mg/kg BW for growing sheep, and the AFRC (56) suggested 40 mg/kg BW, translating to 1.2–1.6 g for lambs in our trial. Our results, ranging from 1.47 to 1.97 g, are consistent with these recommendations and highlight the influence of growth stage, breed, and environment on P requirements.

Estimates of the net P requirement for growth in sheep vary depending on the nutritional system used. Our comparative slaughter experiments determined the net P growth requirement for *Yunnan Semi-fine Wool Sheep* to be 6.19 to 5.34 g/kg EBW for animals weighing between 30 and 40 kg. The NRC (48) recommends a net P growth requirement of 6.0 g/kg BW for sheep. However, these recommendations are based on the ARC (6) figures, and it is acknowledged that advancements in breeding and changes in environmental conditions have altered nutritional requirements. Recent studies by international researchers have investigated the mineral needs of various animal breeds and growth stages, revealing some inconsistencies in their findings. For example, Bellof et al. (49) reported a net P growth requirement of 7.5 g/kg EBW for German Merino sheep weighing 30–55 kg. Similarly, Gomes et al. (22) found net P growth requirements ranging from 8.8 to 9.0 g/kg EBW for 5–20 kg Saanen lambs, while Araújo et al. (50) reported net P growth requirements of 7.86–8.74 g/kg EBW for Moxoto goats weighing 15–25 kg. In China, the net P growth requirement for meat sheep weighing 25–35 kg was estimated to be between 5.04 and 5.35 g/kg EBW (10). Our findings are in close agreement with those of Chinese meat sheep studies and the NRC recommendations but are lower than the values reported by Bellof, Gomes, and Araujo et al.

## 5 Conclusion

The effects of varying dietary phosphorus (P) levels on growth, slaughter performance, and digestive metabolism in *Yunnan Semi-fine Wool Sheep* during the growing period were systematically investigated. Phosphorus levels influenced the metabolism of phosphorus, calcium, protein, and energy, as well as the apparent digestion rates of acid detergent fiber. Based on the experimental conditions, a dietary P level of 0.40% was identified as optimal. Using the minimum endogenous and comparative slaughter methods, the P requirements for maintenance and growth were determined to be 0.06 g/kg EBW and 6.19 to 5.34 g/kg EBW, respectively. These findings support strategies aimed at optimizing P intake to meet maintenance and growth needs while potentially reducing excess phosphorus use in livestock nutrition.

## Data Availability

The raw data supporting the conclusions of this article will be made available by the authors, without undue reservation.
